# Autistic and non-autistic individuals show the same amygdala activity during emotional face processing

**DOI:** 10.1186/s13229-024-00582-9

**Published:** 2024-01-10

**Authors:** Benedikt P. Langenbach, Dominik Grotegerd, Peter C. R. Mulders, Indira Tendolkar, Jasper van Oort, Fleur Duyser, Philip van Eijndhoven, Janna N. Vrijsen, Udo Dannlowski, Zarah Kampmann, Katja Koelkebeck

**Affiliations:** 1 Department of Psychiatry and Psychotherapy, Faculty of Medicine, LVR-University-Hospital Essen, University of Duisburg-Essen, Virchowstr. 174, 45147 Essen, Germany; 2 Center for Translational Neuro- and Behavioral Sciences, University Duisburg-Essen, Hufelandstrasse 55, 45147 Essen, Germany; 3 Institute for Translational Psychiatry, University of Münster, Albert-Schweitzer-Strasse 11, 48149 Munster, Germany; 4 Department of Psychiatry, Radboud University Medical Center, Geert Grooteplein Zuid 10, 6525 GA Nijmegen, The Netherlands; 5 Donders Center for Cognitive Neuroimaging, Kapittelweg 29, 6525 EN Nijmegen, The Netherlands

**Keywords:** Autism, Amygdala, Face processing, Emotion processing, fMRI

## Abstract

**Background:**

Autistic and non-autistic individuals often differ in how they perceive and show emotions, especially in their ability and inclination to infer other people’s feelings from subtle cues like facial expressions. Prominent theories of autism have suggested that these differences stem from alterations in amygdala functioning and that amygdala hypoactivation causes problems with emotion recognition. Thus far, however, empirical investigations of this hypothesis have yielded mixed results and largely relied on relatively small samples.

**Methods:**

In a sample of 72 autistic and 79 non-autistic participants, we conducted a study in which we used the Hariri paradigm to test whether amygdala activation during emotional face processing is altered in autism spectrum disorder, and whether common mental disorders like depression, ADHD or anxiety disorders influence any potential alterations in activation patterns.

**Results:**

We found no evidence for differences in amygdala activation, neither when comparing autistic and non-autistic participants, nor when taking into account mental disorders or the overall level of functional impairment.

**Limitations:**

Because we used one basic emotion processing task in a Dutch sample, results might not generalise to other tasks and other populations.

**Conclusions:**

Our results challenge the view that autistic and non-autistic processing of emotional faces in the amygdala is vastly different and call for a more nuanced view of differences between non-autistic and autistic emotion processing.

## Background

Autistic individuals often show, among other traits, pronounced alterations in their recognition and display of emotions [46]. Frequently, this results in difficulties in social interactions, which may in turn cause substantial individual suffering. Subcortical structures such as the amygdala have been shown to be crucial for both emotion recognition and unconscious processing of emotional faces [15]. In line with the amygdala theory of autism [5], which assumes amygdala alterations to be (partly) responsible for differences between autistic and non-autistic individuals, several studies showed significantly decreased amygdala activation in autistic individuals when presented with emotional stimuli in functional magnetic resonance imaging (fMRI) [4, 20]. Other studies, however, have found increased amygdala activation [28, 36, 45] and yet others have shown no difference at all [21] or no difference in local activation, but differences in functional connectivity [37]. A recent meta-analysis on face processing in autism (including several tasks using emotional stimuli) found a lower activation of the amygdala during face processing to be the only statistically significant alteration in autistic participants [10]; the authors, however, used two different statistical ways to compute the meta-analysis and found significant results in the amygdala only with one of them. A meta-analysis on 13 studies investigating *emotional* face processing found a reduced activation of the amygdala in autism spectrum disorder (ASD), but only when restricting the analysis to studies comparing emotional faces to non-faces, not when comparing emotional faces and non-emotional faces or when combining the two types of comparisons [2], suggesting that the differences in amygdala activation might stem from the faces per se, not the portrayed emotion.

There are several possible explanations for these incongruent results. First, it might be a question of which paradigm was used to elicit an amygdala response. For example, Baron-Cohen et al. [5] used an emotion recognition task using pictures of eyes as stimuli, Weng et al. [45] employed an emotion recognition task using faces, and Kleinhans et al. [28] used a task that involved emotional faces, but did not require participants to judge the portrayed emotions. It might be possible that differences in amygdala activation only appear when fixation of the eyes is required (see also [30]), or that recognising an emotion elicits a different activation than the one caused by merely looking at emotional faces.

Second, the inconclusive results could be due to small sample sizes in some of the studies. For example, Baron-Cohen (2000) had six autistic participants, Monk et al. [36] had twelve autistic participants and Weng et al. [45] tested 22 autistic participants. Indeed, among the 36 studies included in Costa et al.’s meta-analysis [10], only four had more than 30 autistic participants (the average was 18); among the nine studies included in the relevant sub-analysis by Aoki et al. [2], only one had more than 30 autistic participants (the average was 20). While these sample sizes are not unusually small, they do make statistical artefacts more likely.

Third, it might be possible that results were affected by the occurrence of comorbid mental disorders: indeed, a recent meta-analysis on comorbidities in ASD put the life-time prevalence of depression and anxiety disorders at as high as 37% and 42%, respectively [24]. For these disorders, there is some evidence showing altered amygdala activation in reaction to emotional stimuli [17, 32, 39], even though results remain inconclusive [41]. Additionally, previous research suggests that the level of amygdala activity towards emotional faces might be moderated by the level of social anxiety [29].

Another relevant comorbidity might be Attention Deficit Hyperactivity Disorder (ADHD). Comorbidity of ASD and ADHD is common [1] and some alterations in amygdala activation during the processing of emotional stimuli have been reported for ADHD as well [44].

In the study at hand, we wanted to address these issues and tested, in an adequately sized sample, whether autistic and non-autistic participants differ in their amygdala activation during the processing of emotional faces and whether results are influenced by relevant comorbidities, as previous studies have shown the importance of including comorbidities in large-scale analyses [8, 14]. To this end, we analysed functional magnetic resonance imaging (fMRI) data of 72 autistic participants with different types of comorbidity and 79 healthy non-autistic participants. To elicit an amygdala response, we chose a face-matching task [22]. In this task, participants are presented with emotional faces or geometrical shapes and have to indicate which of two faces/shapes at the bottom of the screen is identical to the one at the top of the screen (see “Materials and methods” section). There is ample research showing the task reliably elicits amygdala activation [12, 16, 26, 27]. We included ADHD, depression and anxiety as covariates into our analysis and additionally controlled for severity of patients’ autistic traits (see “Materials and methods” section).

## Materials and methods

### Sample

Data were taken from the Mind-Set sample [43] and collected between 2016 and 2020 at an academic outpatient clinic in Nijmegen, the Netherlands. After data exclusion (see below for details), we analysed the data of 72 autistic participants (23 female) and 79 non-autistic participants (42 female). The mean age was 37 (SD: 15, range: 18–70) and 33 (SD: 12, range: 18–74) for the autistic group and the comparison group, respectively. When comparing the two groups using Welch’s t-test, no statistically significant age differences emerged, *t*(139) =  − 1.4439, *p* = 0.151. For nine autistic participants and one non-autistic participant, age had not been recorded and was substituted with the mean age of their group for all analyses. Of the autistic participants, 17 had a depression, 25 an anxiety disorder, 12 social phobia and 27 ADHD. Some participants had both a depression and an anxiety disorder, so that in total, 36 autistic participants had a depression and/or an anxiety disorder. Autistic participants were recruited at the outpatient unit of the psychiatric department of the Radboud University Medical Center. Exclusion criteria were intellectual disability, mutism, inadequate command of the Dutch language, IQ estimate below 70, psychosis, and inability to record an MRI (e.g. because of metal transplants) or diseases of the central nervous system resulting in permanent sensorimotor or neurocognitive impairments. The autistic participants underwent a 3-h clinical examination including psychiatric, biographical and somatic anamnesis and structured clinical interview. Non-autistic participants were individuals with no current or past psychiatric diagnosis, as confirmed in a standardised diagnostic interview. Examinations were conducted by trained clinicians. At the end of the examination, the senior clinician assessed eligibility based on the DSM-5 classification. For all measures and a detailed description of the recruitment and assessment, see Eijndhoven et al. [43].

### MRI acquisition and processing

#### Image acquisition

MRI data were collected at the Donders Centre for Neuroimaging using a 3T Siemens Magnetom Prisma system with a 32-channel head coil. T2*-weighted echo planar images with blood-oxygen-level-dependent contrast were acquired during the emotion processing task (repetition time [TR] = 1000 ms, echo time [TE] = 34 ms, slicing: interleaved ascending, voxel size: 2.0 × 2.0 × 2.0 mm, flip angle: 60°). In addition, anatomical images were acquired on the same scanner using a T1-weighted MP-RAGE sequence (TR = 2300 ms, TE = 3.03 ms, voxel size: 1.0 × 1.0 × 1.0 mm, flip angle: 8°, GRAPPA acceleration factor: 2).

#### Image processing

Functional data were processed using statistical parametric mapping software (SPM8, Welcome Department of Cognitive Neurology, London, UK; http://www.fil.ion.ucl.ac.uk/spm). During pre-processing, data were motion‐corrected, spatially normalised to standard Montreal Neurological Institute (MNI) space and smoothed (Gaussian kernel, 8 mm full-width at half-maximum (FWHM)). Experimental conditions (faces, shapes) were folded with the canonical haemodynamic response function and entered in General linear models to create individual statistical parametric maps for the contrast of interest faces > shapes. A high-pass filter of 128 s was applied to remove low-frequency noise. Movement parameters were entered as nuisance regressors. After quality control, eight subjects had to be excluded from the fMRI analyses due to excessive head movement (exclusion criterion 3 mm/3°).

### Emotion paradigm

In the MRI scanner, participants performed a short version of a widely used emotion processing paradigm that elicits robust amygdala activity [22]. Participants were presented with three pictures (one target picture above the other two) and had to indicate which picture from the bottom row matched the identical target picture. In the face condition, the stimuli consisted of faces expressing anger or fear. In the shapes condition, the stimuli consisted of ellipses that were oriented either vertically or horizontally. Participants used their right index and middle finger to give their answer. Within about 3 min, participants completed two faces and three shapes blocks, consisting of six trials each and lasting 30 s each. As part of the MIND-set experiments, participants completed additional tasks which are described elsewhere [43].

### Questionnaires

Participants filled in the Dutch version of the Autism Questionnaire (AQ; [23]) and the WHO Disability Assessment Schedule 2.0 (WHODAS; [42]). From the WHODAS, the subscale “Getting along” was used, which assesses participants’ difficulties with, for example, maintaining a friendship or dealing with strangers such as shopkeepers or service personnel.

### Statistical analyses

The analysis of behavioural data was carried out in R [38], all other statistical analyses were carried out with SPM8 [3]. For the behavioural analyses, we first calculated the rate of correct matches per condition (face/shape) for each participant and then compared the groups using an ANOVA. For the analysis of the fMRI-data, we focussed on an amygdala-region-of-interest (ROI), defined anatomically using the WFU PickAtlas [34]. We first checked whether the task elicited amygdala activation (across participants) using a one-sample t-test on the first-level contrast maps to compare processing of faces vs. shapes. In a next step, we used a two-sample t-test to compare amygdala activation between autistic and non-autistic participants and subsequently used ANOVAs to compare different groups of autistic participants based on psychiatric comorbidity (see below). In separate ANOVAs, we included diagnosis and questionnaire scores (AQ, WHODAS) as covariates when comparing non-autistic and autistic participants. To corroborate non-significant results, we used JASP [25] to calculate Bayesian analysis and for figures. Finally, we calculated a psychophysiological interaction to investigate functional connectivity. Age and gender were included as covariates in all analyses. Whenever we use the term “no statistically significant differences” in the following sections, we are referring to the very liberal criterion of *p* < 0.001 (uncorrected).

### Community involvement

An established patient advisory board, including autistic advisors, gave input for design and execution of the Mind-Set Study, of which this project is a part.

## Results

### Behavioural results

In a first step, we analysed participants’ performance in the face-matching paradigm using an ANOVA to compare autistic and non-autistic participants and including age and gender as covariates. The hit rate in the face condition was 0.865 (SD: 0.196) for the autistic participants versus 0.871 (SD: 0.150) for non-autistic participants. For the shape condition, the hit rate was 0.901 (SD: 0.163) for the autistic participants versus 0.913 (SD: 0.124) for non-autistic participants. There was no statistically significant difference between the autistic and non-autistic participants, either for the face condition, *F*(1,149) = 0.093, *p* = 0.761, or for the shape condition,* F*(1,149) = 0.25, *p* = 0.592.

### Proof of principle

To check whether the task did indeed elicit amygdala activity, we calculated a one-sample t-test using the first-level contrast of faces > shapes over all participants. As expected, we found a strong bilateral activation of the amygdala when participants saw emotional faces compared to shapes (peak MNI coordinates − 18 − 6 − 18, *t* = 20.42, *p*_FWE_ < 0.001 and 22 − 4 − 18, *t* = 20.12, *p*_FWE_ < 0.001, FWE-corrected at the cluster level for an amygdala-ROI).

### Comparison of autistic and non-autistic participants

**Fig. 1:**
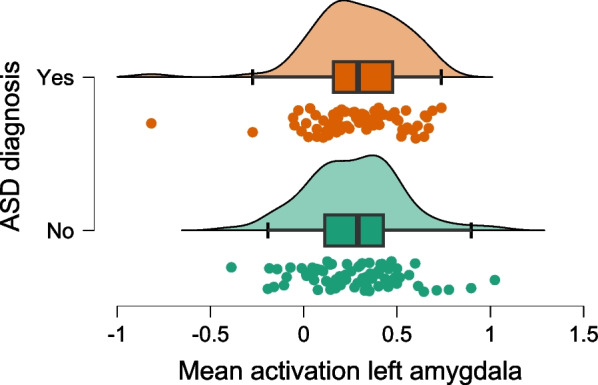
Mean activation in the left amygdala. The figure depicts the extracted values for the mean activation per participant alongside a density blot and a boxplot (showing 1st quartile, median, and third quartile; whiskers extend up to 1.5 times the interquartile range)

**Fig. 2:**
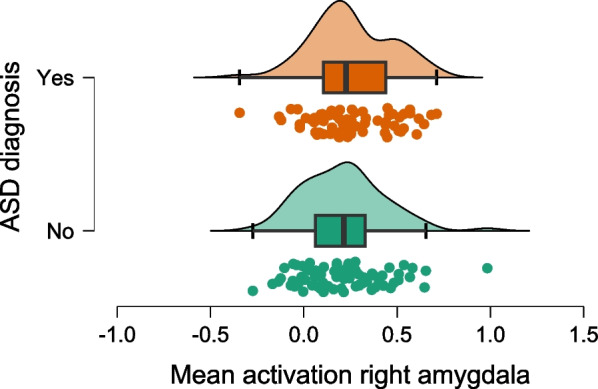
Mean activation in the right amygdala. The figure depicts the extracted values for the mean activation per participant alongside a density blot and a boxplot (showing 1st quartile, median, and third quartile; whiskers extend up to 1.5 times the interquartile range)

Next, we compared the autistic and non-autistic participants using a two-sample t-test and the same contrast as above, but without any regard for comorbidities. There were no significant differences in brain activation in the amygdala ROI, even when applying the very liberal criterion of *p* = 0.001 (uncorrected), see Figs. 1 and 2 for a graphical depiction including individual participant data.

### Influence of comorbidities

Because autistic individuals frequently suffer from comorbid disorders, we then used ANOVAs to compare three groups of participants: non-autistic participants, autistic participants with comorbid disorders and autistic participants without comorbid disorders. We calculated separate ANOVAs for the following comorbidities: ADHD, current depressive episode, current social phobia, current anxiety disorder (any), current anxiety disorder and/or current depression (with the last group comprising patients with either anxiety disorder or depression as well as patients with both disorders) for the number of participants fulfilling each criterion. We then used *F*-contrasts to compare the non-autistic participants to autistic participants with comorbidities, to compare the non-autistic participants to autistic participants without comorbidities and to compare the autistic participants with comorbidities to those without. Again, there were no statistically significant differences in brain activity in the amygdala-ROI.

To corroborate the results, we reran a two-sample t-test comparing the autistic and non-autistic participants, but one time including ADHD as a covariate, then using (any) current anxiety disorder and depression as covariates. Again, there were no statistically significant differences using the same criteria as before.

### Controlling for AQ und WHODAS

In a last step, we wanted to check whether the degree of disability and the strength of autistic symptoms influenced the results. As before, we compared activation between autistic and non-autistic participants, this time using the AQ and the WHODAS’ “Getting-Along”-subscale as covariates (in separate analyses). There were no statistically significant differences. Finally, we analysed whether the AQ score was connected to amygdala activity and calculated a multiple regression with AQ as predictor and age, gender and autism diagnosis (yes/no) as covariates. Once again, there was no statistically significant connection between AQ and amygdala activity.

### Bayesian analyses

In a frequentist statistical framework, the absence of evidence for an effect must not be interpreted as evidence for the absence of an effect. Thus, to corroborate our results, we also employed Bayesian statistics and calculated the Bayes’ Factor BF_01_, stating the predictive probability of the alternative hypothesis (no effect) over the hypothesis that there is an effect. For example, BF_01_ = 2 would mean that it is twice as likely to obtain our data if there was no effect. A common interpretation is that a BF_01_ > 3 indicates moderate evidence for the alternative hypothesis, and BF_01_ > 10 indicates strong evidence. To this end, we first extracted the mean activation per participant in the left and right amygdala clusters, respectively, and then used the extracted data to calculate the Bayesian analyses using JASP. Visual inspection of a quantile–quantile-plot showed that normality of residuals can be assumed.

We first calculated a Bayesian t-test comparing autistic to non-autistic participants, and obtained a BF_01_ = 2.825 and BF_01_ = 4.960 for the right and left amygdala, respectively. Thus, our data were 2.825 and 4.96 times more likely to be observed under the null hypothesis.

Next, we wanted to include gender and age as covariates, as is standard in neuroscientific analysis. To this end, we ran an ANCOVA with ASD diagnosis as predictor and age and gender as covariates. ASD diagnosis yielded a BF_exclusion_ = 3.008 (for the right amygdala) and 4.930 (for the left amygdala), indicating that the data are 3.008 and 4.93 times more likely under models that exclude ASD diagnosis as predictor than under models that include it. Similarly, comparing a model only including age and gender with a model including age, gender, and ASD diagnosis, resulted in a BF = 3.561 (for the right amygdala) and BF = 4.791 (for the left amygdala), favouring the models without ASD diagnosis over the one with ASD diagnosis. Due to the nature of the statistical estimations implemented in JASP (e.g. Markov chain Monte Carlo), the exact results will vary slightly. However, error percentages remained below 0.05% for the t-tests and below 1.6% for the ANCOVAs, and therefore well below the cut-off of 10% that’s proposed by the developers of JASP.

All in all, the Bayesian results support the notion that there is no difference between autistic and non-autistic participants in terms of amygdala activation during our task.

### Functional connectivity

A recent study investigating 16 autistic and 21 non-autistic women showed pronounced differences in the functional connectivity of the amygdala to various other brain areas between the two groups [37]. Thus, we calculated a psychophysiological interaction (PPI; [18]) with the bilateral amygdala as seed region. Put simply, a PPI is the mathematical interaction of a given task design (e.g. experimental condition vs. control condition) and the time series of a given brain area (the physiological variable). This term is then used to predict brain activation in other brain areas than the chosen seed area. If the term has predictive power beyond the main effects of the psychological and physiological variables alone, this indicates a functional (i.e. task-dependent) connectivity between those regions. Here, we used SPM8’s built-in function to calculate a PPI.

With the newly calculated PPI with the amygdala as seed region, we found no statistically significant differences in functional connectivity between autistic and non-autistic participants, with the lowest *p*_FWE_ = 0.609. When including ADHD as covariate, no statistically significant results emerged, either.

## Discussion

In a large sample of 72 autistic and 80 non-autistic participants, we tested the hypothesis that amygdala activation during processing of emotional faces is altered in autism spectrum disorder. While our paradigm elicited strong activation of the amygdala across the entire sample, no differences were found between autistic and non-autistic participants. These results were unaffected by common comorbidities of autism spectrum disorder, i.e. depression, anxiety disorders and ADHD. Additionally, the results were unaffected by participants’ AQ score and their impairment in interacting with others according to the WHODAS.

These results may be surprising, especially considering the prominent view that alterations in emotion processing and social processing in ASD are largely due to changes in the development and functioning of the amygdala [5]. However, as noted above, previous functional studies have yielded mixed results, calling this hypothesis into question [4, 20, 21, 29, 36, 45]. On a structural level, a comparatively larger amygdala volume has been described in ASD, but also reduced amygdala volume and no volume change at all. A recent meta-analysis found evidence of structural changes only in the right amygdala, not in the left, but reported high heterogeneity between studies and a possibility for publication bias [31].

Our results seem to indicate that in the amygdala processing of emotional faces, there is no difference between autistic and non-autistic individuals, questioning the idea that altered emotion processing of basic facial expressions in autism is due to changes in the amygdala. There are, however, several aspects to consider when evaluating our results. First, one might wonder whether our results are task-dependent or whether they generalise to other tasks (or naturalistic settings). In the task at hand, participants did not have to recognise or process the emotion in order to solve the task, and emotion processing and emotion recognition was purely incidental. It might be speculated whether differences in amygdala activity would only emerge in a task that does require participants to recognise (i.e. name) an emotion. This idea gets further support from the fact that autistic and non-autistic participants did not differ in their performance of the task. It might be possible that neural differences only emerge in a task where also behavioural differences are observable, such as the Reading The Mind In The Eyes Task [6]. While this idea certainly justifies further empirical examination, we do not believe that it devalues our results, for several reasons:

First, the task used in this study is known to elicit a strong amygdala response (and does so in our sample, too). If alterations in emotion recognition in ASD were actually due to an altered functioning of the amygdala, it would be fairly surprising to see no differences in amygdala activation when confronted with emotional faces. Second, previous studies have reported altered amygdala activation in autism using tasks that do not require emotion recognition, and even during subliminal presentation of faces [21]. Based on these findings, one would also expect an altered amygdala response in the task at hand, yet this was not the case. Third, even in fMRI-studies that did require emotion recognition, results were mixed, with some studies showing increased amygdala activity in ASD [11], others showing decreased activity [5] and yet others reporting no difference [13]. Still, future studies might want to corroborate our findings using a different task.

It should be noted that some studies reported a stronger activation of the amygdala when participants had to fixate on the eyes [33] and it has been proposed that subcortical hyperactivation could be the reason why eye-contact is often experienced as highly aversive for autistic individuals [19]. Thus, future studies might want to control for this effect.

It should also be noted that the amygdala activation commonly observed in the face-matching task might not necessarily be due to the emotional nature of the stimuli: Wright and Liu [47] observed a similar amygdala activation during the matching of neutral faces and conclude that amygdala activity is partly due to relevance detection, not emotion processing. While this is an interesting observation about the role of the amygdala (and has helped to get past a simplistic view of the amygdala as “emotional hub”), it does not devalue the results at hand. Rather, one might ask whether “neutral” faces truly exist or whether a blank face looking at somebody does convey an emotional message. Indeed, it has been shown that neutral faces are perceived as negative by healthy individuals [40] and, at least in anxiety disorders, might suffice as a threatening stimulus [9]. More importantly, however, our results might raise doubt about previous findings that regard amygdala alterations as a cause for differences in emotion recognition. In our view, a more nuanced view of the amygdala function is in line with our finding, rather than contrary to it. Additionally, it might be wise to investigate brain connectivity and networks rather than isolated brain areas, e.g. analysing connections between the amygdala and the medial prefrontal cortex or temporo-parietal junction.

Finally, one might criticise that our stimuli consisted only of non-autistic individuals portraying emotions, and wonder, in line with the “double empathy hypothesis” [35], whether activation would be different if emotions had been portrayed by autistic individuals. However, since it is known that emotions portrayed by autistic individuals are equally poorly understood by non-autistic and autistic participants [7], it seems unlikely that this would alter amygdala activation.

## Limitations

Given the varied nature in which ASD presents itself, one can speculate whether our results hold for different subgroups of patients. While we did control for the overall level of impairment and AQ values, one might wonder whether (for example) patients with stronger problems with emotion recognition might show different patterns of amygdala activity.

Similarly, there is a plethora of experimental and clinical tasks aiming to measure emotion processing. It would be presumptuous to assume that no difference in amygdala activity can be expected in all of those tasks, and whether our findings generalise to more complex emotion processing tasks is an open empirical question.

Finally, it is known that research findings in both psychiatry and social neuroscience can differ substantially between different samples, e.g. across different countries and ethnic backgrounds. Thus, our findings might not replicate cross culturally.

## Conclusion

In sum, we found no evidence for altered amygdala activity during processing of emotional faces in ASD, adding to previous research on this topic using smaller sample sizes that had come to inconclusive results. While it is possible that diverging results are partly due to task-specific effects, our data do not support the still ubiquitous idea that autistic alterations in processing of emotional faces are due to alterations in the amygdala, and theories relying on this idea to explain autistic symptoms should be re-examined in future studies. Rather, our results might help to move away from viewing emotion processing in autistic individuals as inherently disordered and pave the way to a more nuanced understanding of the differences between autistic and non-autistic emotion processing. Additionally, our study might serve as a reminder that results from studies with a low number of participants should be interpreted more cautiously.

## Data Availability

The data sets generated or analysed during this study is not publicly available due to privacy reasons but are made available for researchers within the digital research environment upon reasonable request to the corresponding author and approval of the steering board of the MIND-SET study group.

## Abbreviations

AQ: Autism questionnaire
ADHD: Attention deficit hyperactivity disorder
ASD: Autism spectrum disorder
fMRI: Functional magnetic resonance imaging
WHODAS: World Health Organisation Disability Assessment Scale
